# Bacteriophage T4 and its relatives

**DOI:** 10.1186/1743-422X-7-293

**Published:** 2010-10-28

**Authors:** Jim D Karam, Eric S Miller

**Affiliations:** 1 Department of Biochemistry, Tulane University Health Sciences Center, 1430 Tulane Avenue, New Orleans, LA, USA; 2 Department of Microbiology, Campus Box 7615, North Carolina State University, Raleigh, NC 27695, USA

## 

In the coming months Virology Journal will publish a number of authoritative reviews about the biochemistry, structural biology and genomics of the bacteriophage T4 and the T4-related phages. Phage T4 is one of the most extensively investigated viruses and has been the central focus of several monographs and reviews over the last 25 years. Its popularity among experimental biologists is related to the ease with which this phage and some of its relatives can be propagated in widely available nonpathogenic laboratory strains of *Escherichia coli *and the diversity of experimental approaches that can be used to analyze its DNA genome and the RNA and protein products it encodes. The T4 biological system is amenable to investigation by genetic, phylogenetic, biochemical, biophysical, structural, computational and other tools.

Advances in T4 science have paralleled advances in Molecular Biology since the birth of this interdisciplinary field around the middle of the 20^th ^Century [1,2]. Such seminal discoveries as the chemical nature of the gene, the existence of messenger RNA, how the genetic code is read, how genes determine protein structure, how DNA is replicated by multicomponent protein machines and many other findings that have become integral to our current understanding of basic molecular mechanism in biology have typically involved important contributions from the T4 and T4-related experimental systems. The last monograph to comprehensively review all aspects of the molecular biosciences of the T4 virus was published in 1994 [3]. Since that time, the field of Molecular Biology has undergone considerable transformation, particularly as a consequence of advancements in the methods for sequencing microbial and eukaryotic genomes and using DNA sequence data for novel experimental designs that have yielded numerous rewards in resolving biological mysteries and stimulating the growth of biotechnology. The review series to be published in Virology Journal will emphasize advances and seminal discoveries in four major areas of T4 research: Genomics, Gene Expression, DNA Replication and Phage Morphogenesis.

## Genomics

Phages that share an evolutionary history with T4 are highly abundant in nature and can be detected by simple plating techniques using a diversity of bacterial genera or species as hosts. Over the last several years, advances in DNA sequencing technologies have made it possible to analyze the genomes of a large number of these phages, including both close and distant phylogenetics relatives of T4. The sequence database for these T4-like phages is a rich source of insights into the mechanisms of genome replication, expression, packaging and diversification in evolution. In many cases, the experimental systems and genetic tools to test these insights are available. In a review entitled ***Genomes of the T4-related phages as windows on microbial evolution, ***V. Petrov, S. Ratnayaka, J. Nolan, E. Miller and J. Karam summarize the genome sequence information currently available in databases for more than 40 T4 relatives that represent a wide array of specificities to bacterial hosts. Genomes have been sequenced from T4-related phages that infect strains of *Enterobacteria, Aeromonas, Acinetobacter, Klebsiella, Pseudomonas, Vibrio *and marine *Synechococcus *and *Prochlorococcus*. Comparisons between these genomes reveal a high degree of genetic diversity around a conserved core of genes that determine the replication, temporal expression and packaging (phage morphogenesis) of a specifically designed dsDNA viral chromosome. The review draws parallels between the diversity of this large and mosaically organized group of phage genomes and the type and extent of diversity that is being observed within groups of prokaryotic and eukaryotic genomes in general. The broad natural distribution of the T4-related phages includes the largest ecosystem of our planet, the marine environment. A review by M. Clokie, A. Millard and N. Mann (***T4-related phages of the marine ecosystem*) **focuses on the comparative genomics and other studies of T4 relatives that infect cyanobacteria, particularly the genera *Synechococcus *and *Prochlorococcus*. The results of these studies have implications about the possible roles of the T4-related cyanophages as traffickers of bacterial genes, including genes involved in photosynthesis, and the potential impact of host-phage interactions on control of the marine ecology. A remarkable feature of T4-related genomes, irrespective of the host range or geographical origin, is the abundance and diversity of mobile DNA elements that have colonized this group of phages in evolution. Studies with phage T4 were among the first to show the natural existence of mobile introns in the prokaryotic world and to elucidate the mechanisms of intron mobility through the action of homing endonucleases [4]. Almost every category of homing endonuclease genes has been detected in the group of T4-related genomes sequenced so far. These genes can exist inside as well as outside of introns and the homing enzymes they produce can mobilize a diversity of DNA sequences laterally between genomes of the T4 family of phages[5]. In the article entitled ***Mobile DNA elements of the T4-related phages***, D. Edgell, E. Gibb and M. Belfort review the major progress that has been made over the last 15 years in our understanding of the structures, mechanisms of action and physiological roles of these mobile elements and their potential impact on phage and microbial evolution.

## Gene expression

Temporal gene expression constitutes an important part of the strategies used by all viruses to coordinate the different biochemical processes involved in viral genome replication, genome packaging and the release of new generations of virus. In general, the control of gene expression, which is highly permissive to diversification in the evolution of organisms [6], contributes significantly to the adaptation of viruses to new physiological conditions such as the encounter of these infectious genetic entities with new potential hosts. The T4-related phages exhibit many examples of such diversification. In three reviews that deal with the control of T4 gene expression, the authors discuss advances in research on the structures and functions of the phage-encoded proteins that determine the temporal utilization of the phage genome during the different stages of phage development in the bacterial host. The review by D. Hinton (***Control of transcription in the prereplicative phase of T4 development****) *discusses the current state of knowledge about the structures and functions of the protein factors and DNA sites that control phage genome transcription shortly after the entry of the phage DNA into the bacterial host cell. The protein-DNA interactions during the early phase of the phage developmental program set the stage for diverting the host RNA polymerase from transcription of the bacterial genome to transcription of the T4 genes required for phage DNA replication, repair and the other replication-related processes that ultimately ensure the coordination between phage DNA metabolism and phage morphogenesis. Some of the phage-encoded proteins made during the prereplicative phase introduce modifications onto subunits of the host RNA polymerase while others associate with this enzyme and alter the specificity of the transcription apparatus so that expression of the phage genes that determine the structure and assembly of infectious virions is maximized. The review by E. P. Geiduscheck and G. A. Kassavetis (***Transcriptional control during the late phase of T4 development****) *discusses recent progress in the analysis of the structures and biochemical functions of the key proteins, especially gp33, gp45 and gp55, involved in this transition in RNA polymerase specificity from early to late transcription. These proteins play roles in coordinating late transcription with genome replication during the late phase of phage development. The third review, ***Posttranscriptional control of T4 development ***by M. Uzan and E. S. Miller discusses the several biochemical strategies used by phage T4 to control gene expression beyond the level of transcription. This phage encodes a number of proteins that exert differential effects on the utilization of the mRNA for specific phage induced proteins. These strategies include controls over mRNA activation (RNA processing), inactivation (RNA decay and repression of translation) and host ribosome function. All 3 reviews highlight the insights gained from the sequence polymorphism that has been observed among allelic proteins in the databases for T4 relatives.

## DNA replication

T4 encodes all of the proteins required for replication of the phage DNA genome, including the components of a complete DNA replisome and several other proteins that perform important auxiliary functions in the replication, repair and recombination of the genome. The ease with which the T4 system replication system can be analyzed by a wide range of experimental tools and the many similarities this system exhibits to eukaryotic DNA replication machines have made it a widely recognized model for investigators in the DNA replication field. Genetic and biochemical studies of the multi-protein complexes that carry out T4 DNA replication have brought to light the important role that genetic recombination plays in replication [7], elucidated several of the enzyme mechanisms involved at DNA replication forks and provided the generally accepted model for coordination of DNA synthesis between the leading and lagging strands (i.e., the trombone model; [8]). Three reviews will highlight the recent advances in research on the mechanisms of the initiation and DNA chain elongation stages of T4 DNA replication and the structures of the proteins that carry out these processes. A review by T.C. Mueser, J.M. Devos, J.M. Hinerman and K.J. Williams ***(Structural analysis of T4 DNA replication) ***discusses these structures with emphasis on the determinants of biochemical function and by drawing parallels to available structural information about replication proteins from other biological systems. In a review entitled ***Initiation of T4 DNA replication and replication fork dynamics, ***K. N. Kreuzer and R. J. Brister describe recent advances in understanding the interplay between two modes of intiation of T4 DNA replication, one based on the recognition of specific origins on the T4 dsDNA chromosome and one based on the use of the enzymes of homologous recombination to create initiation sites through the invasion of the circularly permuted and terminally redundant phage dsDNA chromosomes by the ends of homologous molecules. Remarkably, like the linear dsDNA chromosomes of eukaryotes, the T4 chromosome harbors multiple sites for intiation (origins) of replication and the review discusses the evidence for differential use of these origins and the take over by recombination-driven initiation during the course of the phage developmental program. Over the last 15 years, the structures of several of the proteins of the T4 DNA replisome and/or homologous proteins from the T4-related phage RB69 have been analyzed at the atomic level by X-ray crystallography. A review by J. Liu and S.W. Morrical (**Assembly and dynamics of the T4 homologous recombination machinery***) *emphasizes advances in research on the structures and biochemical mechanisms of the T4 encoded proteins that support genetic recombination and the initiation of phage DNA replication. As an integral part of the biochemical strategy for generating hundreds of phage genomes per infected cell, the T4-encoded proteins for genetic recombination have evolved to be abundant and highly active and as a consequence, have been accessible for detailed biochemical analysis. They are serving as models for evolutionarily related counterparts in eukaryotes and bacteria.

## Phage morphogenesis

Two reviews in this thematic series focus on the synthesis, structures and assembly of the two major components of the T4 virion, the capsid in which the phage DNA is packaged (T4 heads) and the phage tail and tail fibers, which make it possible for this bacterial virus to recognize its bacterial host and deliver its DNA into the cell. Far from being a hindrance to the experimental biologist, the complexity of the structure of the T4 virion has proven to be a great asset in the elucidation of many biochemical mechanisms that are broadly represented in other systems of viral assembly in cellular hosts. The structure of T4 phage particles, or what is sometimes referred to as the "T4 morphotype", exhibits several features that are conserved among phylogenetic relatives of this phage and that appear to be mimicked by a large number of viruses in nature. A review entitled ***Morphogenesis of T4 heads ***(by V. Rao and L. Black) discusses the new insights that have been gained over the last few years about T4 head assembly from the direct structural analysis of a protein (gp24) related to the major component of the phage capsid (gp23), solid NMR analysis of T4 particles, other biophysical studies and refinements in in vitro assays of DNA packaging. A second review (***Morphogenesis of the T4 tails and tail fibers ***by P. G. Leiman, F. Arisaka, M.J. Van Raiij, A. A. Aksyuk, V. A. Kostychendo, S. Kanamaru and M. G. Rossmann further underscores the impact of recent advances in the structural sciences on understanding of the biochemical processes that underlie the assembly of multi-component nucleoprotein biological structures. The studies reviewed here have led to vivid images of T4 phage particles and the dynamics of phage infection. This review discusses the application of a variety of approaches that determined the structure of the contractile tail of T4 and the broad implications of the findings to the structural organization of other phages with contractile tails.

Some of the reviews in this series will be supplemented by web-based information to be updated as new developments in the field come to light. These supplements and updates will include summaries in the form of PowerPoint charts (including simple animations), Tables or videos that can be used by research scientists and educators alike.

Virology Journal is taking a leading role in facilitating the dissemination of new information in fast-growing areas of phage biology and the series on T4 and its relatives constitutes a first example of the journal's efforts in this regard. We are grateful to Robert F. Garry, Ph.D., Editor in Chief of Virology Journal and Professor at Tulane University for his guidance during the preparation of manuscripts for this thematic series.
